# The effect of needle type (25 G Sprotte vs. Quincke) in spinal anesthesia on the incidence of transient neurologic syndrome: A randomized clinical trial

**DOI:** 10.1002/hsr2.2025

**Published:** 2024-05-01

**Authors:** Somayeh Ziaei, Mitra Yari, Ana Beatriz Pizarro, Parisa Golfam, Alireza Ahmadi

**Affiliations:** ^1^ Anesthesia Department, Emam Reza Hospital Kermanshah University of Medical Sciences Kermanshah Iran; ^2^ Clinical Research Center, Fundación Valle del Lili Cali Colombia

**Keywords:** pain, Quincke, spinal anesthesia, Sprotte, transient neurologic syndrome

## Abstract

**Background and Aims:**

Transient neurologic syndrome (TNS) is a postoperative pain in the back and buttock that can occur after spinal anesthesia. The spinal needle design may have an impact on the occurrence of TNS. We decided to compare the incidence of TNS and related factors between two spinal needle types.

**Methods:**

In this randomized clinical trial, 150 patients aged 18–60 years and American Society of Anesthesiologists (ASA) physical status I who underwent lower abdomen or lower extremity surgeries with spinal anesthesia and supine position were enrolled. They were randomly divided into two groups (25 G Quincke or Sprotte needle) with 0.5% bupivacaine (12.5 mg). After the operation, the patients were asked to report any pain in the lower back, buttock, and thigh areas. A Visual Analog Scale (VAS) was also used to record the severity of the pain.

**Results:**

Overall, 45 patients developed TNS. Twenty‐nine patients in the Sprotte group (38.7%) and 16 patients in the Quincke group (21.3%) developed TNS (*p* = 0.75). More patients in the Sprotte group (25.3%) had severe pain (VAS score of 8–10) when compared with the Quincke group (6.7%). There was no significant difference in TNS symptoms duration between the two groups. In about half of patients (51.9%) in the Sprotte group and 57.3% of patients in the Quincke group, the symptoms resolved after 2–3 h.

**Conclusion:**

Although the incidence of TNS did not differ significantly, patients for whom a Sprotte spinal needle had been used had more severe pain. This suggests that the Quincke needle caused less severe pain.

## 1 INTRODUCTION

Spinal anesthesia, a type of neuraxial block, is used routinely for abdominal and lower extremity operations. This technique has reduced peri‐ and postoperative morbidity and mortality.^1^ On the other hand, several complications may occur after spinal anesthesia.^2^ Hypotension and bradycardia have been reported after spinal anesthesia. Neurological complications such as postdural puncture headaches, sensory or motor nerve dysfunction, and nerve injury have also been reported.^3^ Transient neurologic syndrome (TNS) is a neurologic complication that can occur after spinal anesthesia with a frequency of about 15%.^4^ It is a pain syndrome most often in the back and buttock areas, is usually self‐limited, and could be in the form of dysesthesia. The pain, usually not accompanied by focal neurologic deficits, can be unilateral or bilateral and radiate to the lower extremities. TNS could be mild or severe and usually alleviated after 1 week.^5^

TNS is usually a diagnosis of exclusion.^5^ Some severe conditions such as spinal cord infection and spinal hematoma should be excluded when patients develop pain in the lower back and buttocks after operations. Several risk factors have been described for the occurrence of TNS. Factors such as the local anesthetic used for a spinal block and the patient's position have been relatively strong factors associated with the development of TNS. There is more robust evidence about lidocaine, a typical local anesthetic, and lithotomy position ^6^ as risk factors for TNS.^4^

Another factor that has been noted in the literature as a potential risk factor for TNS is the type of needle and consequent dural trauma. Spinal needles have different designs regarding the number of orifices (single‐orifice or double‐orifice, orifice location, tip design, diameter, etc.). Two standard needles used for spinal anesthesia are 25‐gauge (25 G) Sprotte and Quincke needles. Sprotte needles have single lateral orifices with an elongated pencil point tip. Quincke needles have a cutting bevel and a sharp tip. Although theoretically, it seems that needle design can affect dural trauma and consequently affect the neurologic symptoms following spinal anesthesia, few studies have compared spinal needle types. In a previous study, no difference was observed regarding the incidence of TNS between Sprotte and Quincke needles with lidocaine and bupivacaine. No significant difference was reported between the two needle types.^7^ On the contrary, another study noted that TNS was less frequent with using spinal needles with two orifices (Eldor) than single‐orifice Atraucan needles in 99 American Society of Anesthesiologists Classification of Patients (ASA I‐II) patients.^8^

In this study, we intended to compare the incidence of TNS, the severity of pain, the onset of pain, and the location of pain between Sprotte and Quincke spinal needles.

## 2 MATERIALS AND METHODS

We have employed the latest guidelines for the accurate analysis, reporting, and interpretation of this clinical research.^9^ This was a triple‐blind randomized clinical trial conducted in our university hospital. Inclusion criteria were age range of 18–60 years, ASA physical status I, body mass index (BMI) less than 25 Kg/m^2^, and surgeries of the lower abdomen and lower extremities in a supine position (inguinal hernia, varicocele surgery, and lower limb varicose vein surgery). Exclusion criteria were contraindications to spinal block, infection at the site of spinal anesthesia, coagulation disorders, allergic reactions to local anesthetics, neurologic disorders, opium addiction, chronic low back pain or lower extremity pain, chronic use of analgesics, spinal column abnormalities, and surgery duration >2 h.

The sample size was 150 patients, and they were randomly divided into two groups: one group underwent spinal anesthesia with a 25 G Quincke needle (75 patients), and the other group 25 G Sprotte needle was used (75 patients). Standard monitoring of patients with electrocardiogram, non‐invasive blood pressure, and oxygen saturation was performed. Hydration of the patients was done with lactated ringer intravenous infusion (10 cc/kg) over 20 min. The skin was cleaned with a povidone‐iodine solution of 10% in a sitting position. Spinal anesthesia was performed in the L4–L5 space between the lumbar vertebrae by the midline approach in all patients. The local anesthetic agent injected was 0.5% bupivacaine (12.5 mg; 0.2 mg/sec). A single anesthesiologist performed all anesthesia procedures.

After injecting the anesthetic, the patient was placed in the supine position. The segmental level of the sympathetic block was examined bilaterally by using a cold alcohol swab. Sensory block was assessed using the pinprick method. These assessments were done every 5 min for 20 min and then every 15 min until complete recovery of the sensory and motor block.

The patients were asked to report any lightning pain during the needle insertion. After the operations, the patients were asked to report any pain or burning sensation in the lower back, buttocks, or thigh and document the time of its onset (interval between spinal anesthesia and initiation of pain or dysesthesia) and location. The patients were followed on postoperative days 1 and 3. If the patients had been discharged, they were visited in the anesthesiology clinic to examine and document any complaints. The duration of symptoms was quantified from the spinal anesthesia and the resolution of the pain. When the patient complained of pain, the necessary treatment recommendations were made that included the administration of a nonsteroidal anti‐inflammatory drug (NSAID).

To determine the severity of pain, a Visual Analog Scale (VAS) was used, and the patients were instructed to report their pain severity based on this scale (0 = no pain at all; 10 = most severe pain). Then, pain severity categories were created (1–4, 5–7, and 8–10).

This study have been approved by the Ethics Committee of Kermanshah University of Medical Sciences. We obtained informed consent from all patients. This randomized clinical trial has been approved in the Iranian Registry of Clinical Trials on September 7, 2018 (registration reference IRCT20100101002946N9). First research participant was enrolled on October 20, 2018.

### 2.1 Statistical analyses

Descriptive indices such as frequency, and percentage, expressed the data. To compare categorical and continuous variables between the two groups, the *χ* ^2^ and *t* test were used, respectively. The significance level was defined as 5%. *p* values less than 0.05 were deemed statistically significant. The data have been analyzed using SPSS software (Version 16.0, SPSS Inc.).

## 3 RESULTS

There were 75 patients in each group. Lightning pain was reported in 23 patients in the Sprotte group (30.7%) and 34 in the Quincke group (45.3%); *p* = 0.06. Overall, 45 patients (30%) developed TNS. Twenty‐nine patients in the Sprotte group (38.7%) and 16 patients in the Quincke group (21.3%) developed TNS (*p* value = 0.75).

Table 1 presents the location of the pain reported by the patients. The most common painful locations were the lower back and thigh. As observed, pain in the thigh was more common in the Quincke group.

**Table 1:** Comparison of transient neurologic syndrome (TNS) location in two groups of patients who underwent spinal anesthesia with two different needles.

	Sprotte needle (*n* = 75)	Quincke needle (*n* = 75)	*p* Value
Lower back	22 (33.3%)	26 (38.8%)	*p* < 0.001
Buttock	19 (28.8%)	10 (14.9%)	
Perineum	6 (9.1%)	0 (0%)	
Thigh	14 (21.2%)	27 (40.3%)	
Leg	5 (7.6%)	4 (6%)	

Table 2 summarizes pain severity groups between the two groups. More patients in the Sprotte group had severe pain (25.3%) than in the Quincke group (6.7%). Moderate pain (VAS score 5–7) was relatively similar between the two groups. Fewer patients (21.3%) in the Sprotte group had mild pain than in the Quincke group (48%).

**Table 2:** Comparison of pain severity according to the VAS categories in two groups of patients who underwent spinal anesthesia with two different needles.

VAS score	Sprotte needle (*n* = 75)	Quincke needle (*n* = 75)	*p* Value
1–4	16 (21.3%)	36 (48%)	*p* < 0.001
5–7	40 (53.3%)	34 (45.3%)	
8–10	19 (25.3%)	5 (6.7%)	

Abbreviation: VAS, Visual Analog Scale.

In half of the patients in the Sprotte group, TNS started on postoperative day 2 (36 cases, 48%). Eighteen patients (24%) developed TNS symptoms on postoperative day 1, and 21 cases (28%) developed the symptoms on postoperative day 3. In the Quincke group, 4 (5.3%), 31 (41.3%), and 53 (40.3%) patients developed TNS symptoms respectively on postoperative days 1, 2, and 3. Table 3 presents the duration of TNS before resolution. There was no significant difference regarding the duration of TNS symptoms duration between the two groups. In about half of patients (51.9%) in the Sprotte group and 57.3% of patients in the Quincke group, the symptoms resolved after 2–3 h.

**Table 3:** Duration of the transient neurologic syndrome (TNS) in two groups of patients who underwent spinal anesthesia with two different needles.

	Sprotte needle (*n* = 75)	Quincke needle (*n* = 75)	*p* Value
1 h	5 (6.6%)	4 (5.3%)	0.53
2 h	20 (26.6%)	21 (28%)	
3 h	19 (25.3%)	22 (29.3%)	
4 h	15 (20%)	14 (18.6%)	
5 h	6 (8%)	6 (8%)	
6 h	1 (1.3%)	0	

## 4 DISCUSSION

TNS is a relatively common neurologic complication following spinal anesthesia. According to our findings, 30% of the studied patients developed TNS. The incidence rate of TNS reported in the literature varies from 6.4% ^10^ with mepivacaine, 22% with lidocaine,^11^ and 41.38% with bupivacaine ^12^ and also with ropivacaine this syndrome has been reported.^13^ The exact etiologic factors for this syndrome are not apparent. Ischemia of the spinal cord due to stretching or needle trauma and toxic properties of the local anesthetics have been suggested as possible underlying mechanisms for TNS occurrence.^5^ Several factors such as lidocaine concentration, needle type, lithotomy versus supine position, and epidural anesthesia^14^ have been proposed as potential risk factors.^4^ TNS after the supine position has been less frequent than in the lithotomy position.^7^ This is because, during the supine position, normal spinal lordosis is preserved during the surgery. However, normal lordosis is not preserved in the lithotomy position, which can stretch the nerves, tendons, joints, and cauda equina.^15^

The needle type used for local anesthetic injection may have a contributing role in TNS development. Here, we compared two commonly used spinal needles (Sprotte vs. Quincke) regarding the development of TNS, pain severity, and duration. Both groups received bupivacaine, and the surgeries were performed in the supine position. Therefore, these two factors were balanced between the groups. Based on the obtained findings, the incidence of TNS was comparable between the two needle types. Most patients experienced the pain for 2–3 h. However, patients for whom the Quincke needle had been used reported less severe pain. Needle design, its tip, the number of orifices, and even maldistribution of the anesthetic with some older types of needles have been described as risk factors for TNS occurrence.^16^

A previous study^7^ to explore affecting factors in TNS development reported that although lithotomy position (in comparison to supine position) and lidocaine anesthetic (in comparison to bupivacaine) were significantly associated with TNS. On the other hand, needle type (Quincke vs. Sprotte) was not a significant factor in multivariate analyses. According to the results of this study, 41 patients in the Sprotte group (33%) and 58 patients in the Quincke group (46%) were diagnosed to have TNS (*p* = 0.7). The authors also did not report any significant difference in terms of pain severity quantified by a VAS scoring system. The average severity of pain was six in the study, which was alleviated with pethidine administration. This finding is consistent with the results of our study with the higher pain intensity in the score range of 5–7.

Regarding the type of spinal needle, there was no apparent difference between the two types of needles in the incidence of TNS, which is also consistent with our findings. In another study, a single‐orifice spinal needle (Atraucan) was compared with a double‐orifice needle (Eldor) in 99 patients who underwent urinary or prostate surgeries in supine or lithotomy position with lidocaine. The incidence of TNS was significantly higher in the single‐orifice needle (28.8%) versus those who underwent spinal anesthesia with the double‐orifice needle (8.5%); *p* = 0.006. In further analyses adjusted for needle type, position (lithotomy vs. supine), and type of surgery (prostate surgery or other urologic interventions), needle type remained a significant factor for TNS occurrence (*p* = 0.034). The authors concluded that needle design was a significant factor in TNS development.

Here, we used only one local anesthetic (bupivacaine), a single anesthesiologist performed the procedure, and all surgeries were performed in the supine position. This is important in the study's design as all the mentioned factors may influence the incidence of TNS. These design issues were implicated in focusing on the needle type.

In conclusion, although the incidence of TNS and its duration was not statistically significant between the Sprotte and Quincke groups, the pain severity was less severe in the Quincke group. This requires further studies to include different patient populations and local anesthetics to reach more comprehensive conclusions.

## AUTHOR CONTRIBUTIONS

**Somayeh Ziaei**: Conceptualization; supervision; writing—original draft; writing—review & editing. **Mitra Yari**: Supervision; writing—original draft; writing—review & editing. **Ana Beatriz Pizarro**: Writing—review & editing. **Parisa Golfam**: Validation; visualization; writing—review & editing. **Alireza Ahmadi**: Investigation; supervision; writing—review & editing.

## CONFLICT OF INTEREST STATEMENT

The authors declare no conflict of interest.

## ETHICS STATEMENT

This RCT has been registered in Ethics committee of Kermanshah University of Medical Sciences. Written consent was obtained from participants.

## TRANSPARENCY STATEMENT

The lead author Mitra Yari affirms that this manuscript is an honest, accurate, and transparent account of the study being reported; that no important aspects of the study have been omitted; and that any discrepancies from the study as planned (and, if relevant, registered) have been explained.

## Data Availability

Data will be available in demand.
